# Continuity of Microblade Technology in the Indian Subcontinent Since 45 ka: Implications for the Dispersal of Modern Humans

**DOI:** 10.1371/journal.pone.0069280

**Published:** 2013-07-01

**Authors:** Sheila Mishra, Naveen Chauhan, Ashok K. Singhvi

**Affiliations:** 1 Department of Archaeology, Deccan College, Deemed University, Pune, India; 2 Geosciences Division, Physical Research Laboratory, Ahmedabad, India; University of New South Wales, Australia

## Abstract

We extend the continuity of microblade technology in the Indian Subcontinent to 45 ka, on the basis of optical dating of microblade assemblages from the site of Mehtakheri, (22° 13' 44″ N Lat 76° 01' 36″ E Long) in Madhya Pradesh, India. Microblade technology in the Indian Subcontinent is continuously present from its first appearance until the Iron Age (~3 ka), making its association with modern humans undisputed. It has been suggested that microblade technology in the Indian Subcontinent was developed locally by modern humans after 35 ka. The dates reported here from Mehtakheri show this inference to be untenable and suggest alternatively that this technology arrived in the Indian Subcontinent with the earliest modern humans. It also shows that modern humans in Indian Subcontinent and SE Asia were associated with differing technologies and this calls into question the “southern dispersal” route of modern humans from Africa through India to SE Asia and then to Australia. We suggest that modern humans dispersed from Africa in two stages coinciding with the warmer interglacial conditions of MIS 5 and MIS 3. Competitive interactions between African modern humans and Indian archaics who shared an adaptation to tropical environments differed from that between modern humans and archaics like Neanderthals and Denisovans, who were adapted to temperate environments. Thus, while modern humans expanded into temperate regions during warmer climates, their expansion into tropical regions, like the Indian Subcontinent, in competition with similarly adapted populations, occurred during arid climates. Thus modern humans probably entered the Indian Subcontinent during the arid climate of MIS 4 coinciding with their disappearance from the Middle East and Northern Africa. The out of phase expansion of modern humans into tropical versus temperate regions has been one of the factors affecting the dispersal of modern humans from Africa during the period 200–40 ka.

## Introduction

The period between ~200 ka when the earliest modern humans are found in Africa [1] and ~ 40 ka when the last archaic populations disappeared [2], is complex and variable. Hominins had occupied multiple continents and climatic zones during the Lower Pleistocene, and thereafter began diverging as they adapted to the diverse ecologies over a large geographic range. The evolution of modern humans in Africa [3], Neanderthals in Europe [4] and Denisovans somewhere in Asia [5] is attested too, but probably represents only part of the variability which was developing during the Middle Pleistocene. The Indian Subcontinent, where hominins were continuously present since the Lower Pleistocene [6,7] was probably occupied by yet another archaic species. Genetic evidence suggests that all living people trace most of their ancestry to one of these populations, “modern humans”, who originated in Africa [8]

**Figure 1:**
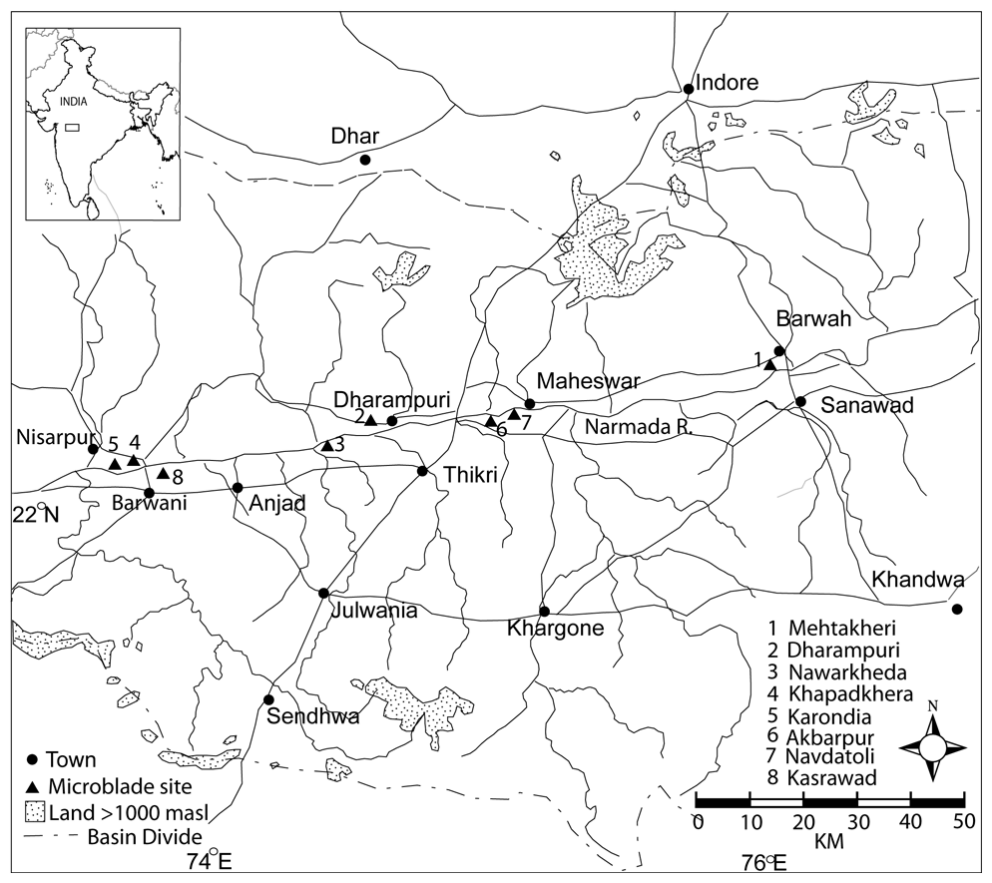
Radiocarbon dated microblade sites in the Nimar region of Madhya Pradesh in the Indian Subcontinent.

The association of microblade technology with modern humans in the Indian Subcontinent is undisputed due to its continuity up to around 3 ka. This is illustrated by eight sites in the Nimar region with 12 radiocarbon ages (Figure 1 Table 1). The site of Mehtakheri is one of these sites. In the Indian Palaeolithic, continuity from the Acheulian to the Middle Palaeolithic and from the earliest microblade assemblages to the Iron Age is seen. Blade technology which has a sporadic but early appearance in the Middle East and Africa [9,10,11,12] is absent from the Indian Acheulian and Middle Palaeolithic. Projectile technology which was an important development in Africa and Europe [13,14] during the post Acheulian period is also virtually absent from the Indian Middle Palaeolithic [15,16]. This suggests that microblade technology is not indigenous to the Indian Subcontinent. Mehtakheri is currently the oldest dated microblade site in the Indian Subcontinent and extends the origin of this technology in the Indian Subcontinent to 44 ± 2 ka based on the weighted average of four dates and ~ 48 ka if the oldest of the dates is accepted as the most accurate as argued below. While microblade technology is associated with modern humans in the Indian Subcontinent, this is not so elsewhere, at least until a much later time period. Modern humans in the Middle East are associated with Middle Palaeolithic technology [17], in Sub-Saharan Africa with the Middle Stone Age [18] and in Southeast Asia [19] and Southern China with core and flake industries [20]. Each of these categories themselves encompasses significant variation.

**Table 1:** Radiocarbon dates for microblade assemblages from the Nimar Region of Madhya Pradesh in the Indian Subcontinent.

	Site name	Latitude Longitude	Lab no	Date (BP)	Cal pal BP	68% range cal BP
1	Mehtakheri	22° 13' 44″ N 76° 01' 36″ E	AA8463	>42,900	> 46,555	>45,028 - 48081
1	Mehtakheri	22° 13' 44″ N 76° 01' 36″ E	A6518	30,106+1040-920	34,380 ± 991	33.389-35371
2	Dharampuri	22° 08' 26″ N 75° 19' 13″ E	BS 286	25,160 ±850	29,975 ± 997	28,977-30972
3	Nawarkheda	22° 06’ 39″ N 75° 14’ 03" E	BS 2243	24,110 ± 820	28971 ± 883	28087-29854
4	Khapadkheda	22° 08' 26″ N 74° 57’ 22″ E	A9446	15,680+440-415	18,860 ± 520	18,340-19380
5	Karondia	22° 04' 09" N 74° 49' 29″ E	BS 1846	16,970 ± 170	20,207 ± 356	20,581-19,840
6	Akbarpur	22° 08’ 95″ N 75° 28’ 07" E	BS 1853	12,390 ± 140	14,610 ± 395	14,214-15005
7	Navadatoli	33° 09’ 29″ N 75° 34’ 51″ E	P 476	4,125 ± 67	4, 669 ± 113	4,556-4782
8	Kasrawad	22° 04’ 27″ N 74° 56’ 15″ E	BS 2242	3,940 ± 120	4,390 ± 180	4,570 - 4,160
8	Kasrawad	22° 04’ 27″ N 74° 56’ 15″ E	BS 2244	3,890 ± 90	4,307 ± 123	4184-4430
5	Karondia	22° 04' 12″ N 74° 49' 29″ E	BS 1872	3,830 ± 70	4,248 ± 113	4.135-4361
7	Navadatoli	33° 09’ 29″ N 75° 34’ 51″ E	P 205	3294 ± 125	3,548 ± 139	3409-3687

Optical dates reported here for Mehtakheri for microblade technology establish that this technology was continuously present in the Indian Subcontinent from 48 ka to 3 ka. This is a longer duration than for any other part of the world. Absence of any precedents for blade technology in the Indian Lower and Middle Palaeolithic make it difficult to consider that this technology developed locally. Indian microblades more closely resemble the African assemblages dating to MIS 4, many of which are blade/microblade based [21,22,23,24] rather than MIS 5 assemblages which include Nubian complex assemblages [25,26,27] and projectiles [28,29] which are extremely rare in the Indian context. In addition to the Howieson’s Poort in South Africa and Mumba rock shelter and Naisiusiu beds in East Africa [30,31] a blade based “transitional” industry is reported from Tarasma in the Nile valley dating to around 60 ka [32,33]. The blade technology at Tarasma evolved from production of Levallois points and does not persist. This industry, unlike the South and East African ones of this time is a blade rather than microblade industry. Presently, the major question in relation to the dispersal of modern humans into the Indian Subcontinent is its timing and associated technology [34]. In one view [35], modern humans entered the Indian Subcontinent with a microblade technology related to that seen between 60–65 ka in South and East Africa. The alternative view is that modern humans entered India earlier, during MIS 5 times with a “Middle Palaeolithic” technology and microblade technology was a later, indigenous development [36,37,38,39,40,41]. Though the presence of microblade technology by 48 ka at Mehtakheri, does not conclusively rule out either of these alternatives, in our view, the first suggestion is more likely as:

1. Blade technology is absent from the earlier Palaeolithic cultures in the Indian Subcontinent so that it is unlikely to be an indigenous development.
2. The transition from Acheulian/Middle Palaeolithic to microblade technology is the only abrupt transition in the Indian Palaeolithic. It seems reasonable to consider that this abrupt change in technology/culture is also related to population change.
3. Stone tool industries dating to MIS 5 in Arabia have no parallel in the lithic industries in the Indian Subcontinent.
4. Stone tool industries younger than MIS 5 in Arabia do not resemble any of the stone tool industries in Africa or Western Asia.
5. MIS 5 contexts in the Indian Subcontinent are associated with Middle Palaeolithic technology that has a clear continuity with the earlier Acheulian technology in India.
6. The Indian microblades have significant resemblances to the earliest microblade sites in South and East Africa dating to between 60–70 ka.

Sharp differences in the stone tool technology of modern humans in the Indian Subcontinent and Southeast Asia exist throughout the Late Pleistocene [19]. This would not be the case if modern humans had reached there from the Indian Subcontinent. Although the evidence is still not conclusive, it appears that modern humans reached Southeast Asia during MIS 5 from a different route and earlier than the Indian Subcontinent. Given the rapidly accumulating evidence for the presence of modern humans in Arabia during MIS 5 [26,27,39,40,41,42,43,44,45,46,47,48], an explanation for their failure to disperse into the Indian Subcontinent at that time, is required. We suggest that the Indian Subcontinent during MIS 5 times was occupied by a population derived from *Homo* *erectus* adapted to the Indian environment from Lower Pleistocene times onwards. This population would be “archaic”, and the Narmada hominin would be ancestral or a representative of it. Competition between Indian archaics and modern humans would have been intense since they were adapted to similar environments. Failure of modern humans to disperse into the Indian Subcontinent during MIS 5 was probably due to their failure to successfully compete with the Indian archaics during a period when the climatic conditions were favourable to both. However during the MIS 4 times, when the desert zones of Africa and Arabia were abandoned and more favourable zones in the Middle East such the Levant and Iran were occupied by Neanderthals, modern humans had more success in entering India and a major change in the Indian Palaeolithic record then occurred. The expansion of modern humans into India therefore coincides with the expansion of Neanderthals into the Middle East at the expense of modern humans and into Central Asia possibly at the expense of Denisovans.

**Figure 2:**
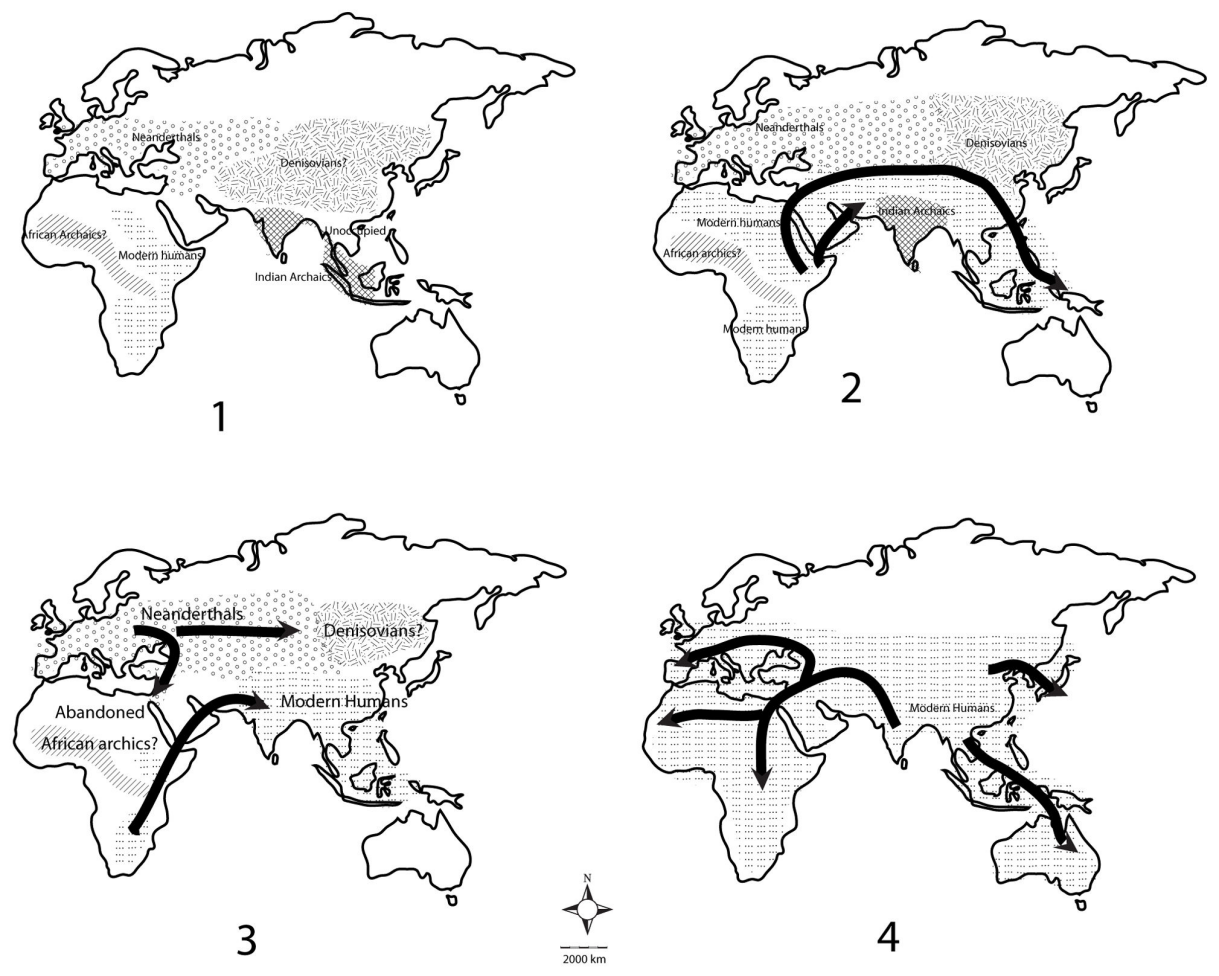
1. MIS 6 : Modern humans and possibly other archaics in Africa. Neanderthals in Europe and Denisovans in Central and Eastern Asia, Indian archaics in the Indian Subcontinent and Sundaland. Equatorial forest zone of SE Asia might not be occupied by hominins. 2. MIS 5: Expansion of modern humans throughout the Tropical and Subtropical zone. Indian archaics retreat from Sundaland which is mostly submerged due to higher sea level. 3. MIS 4: Neanderthal expansion into Middle East at the expense of modern humans and possibly to west. Denisovans might have become extinct at this time. Desert zone of Africa and Asia abandoned by hominins. Archaic Indians disappear with the entry of modern humans into the Indian Subcontinent. 4. MIS 3: Indian Subcontinent a major source for expanding modern humans. Archaic populations disappear with some admixture with modern humans.

Greater resemblance of Indian microblade industries to those in South and East Africa rather than Arabia is probably due to their younger age and their short time of residence in the desert regions which were rapidly becoming hostile to human occupation at the transition from MIS 5 to MIS 4. Figure 2 shows the possible distribution of different populations during MIS 3-6. Further, assemblages younger than MIS 5 (~55 ka) from Wadi Surdud in Yemen [46,47,48] and Jebel Faya in UAE [45] do not resemble any known Palaeolithic entities in either Europe or Africa. While this is interpreted as indicating “local” developments in a period of harsh climate and reduced population densities, it is possible that these industries are more similar to those in the Indian Subcontinent. It is difficult to evaluate this premise as none of the Indian assemblages have been numerically dated to the same time interval. If modern humans were able to spread into the Indian Subcontinent during MIS 4 times, then we should expect that some microblade sites in the Indian Subcontinent should date even earlier than Mehtakheri, as this technology disappeared from South and East Africa around 60 ka or later [22]. This aspect is being currently explored.

## Materials and Methods

### The Mehtakheri Site

**Figure 3:**
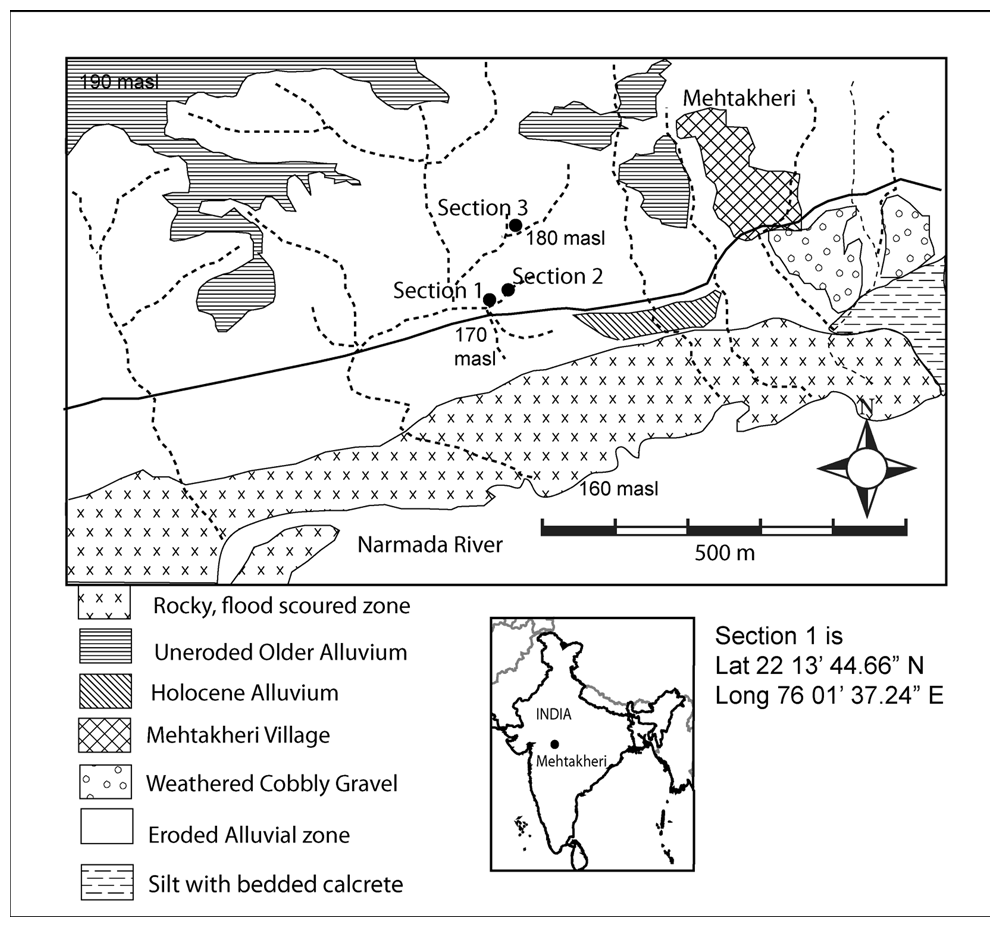
Location of sections at Mehtakheri.

**Figure 4:**
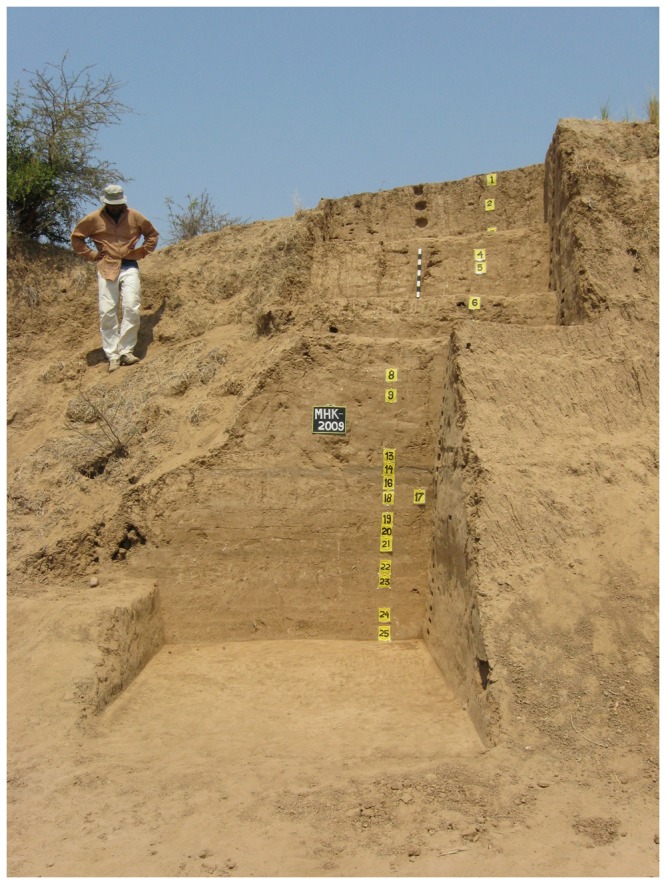
Mehtakheri excavation 2009 showing layers 1-26. Unit 2, containing the artifacts is layers 18-26. Erosional contact between Unit 1 and 2 marked by sand layer above layer 18.

**Figure 5:**
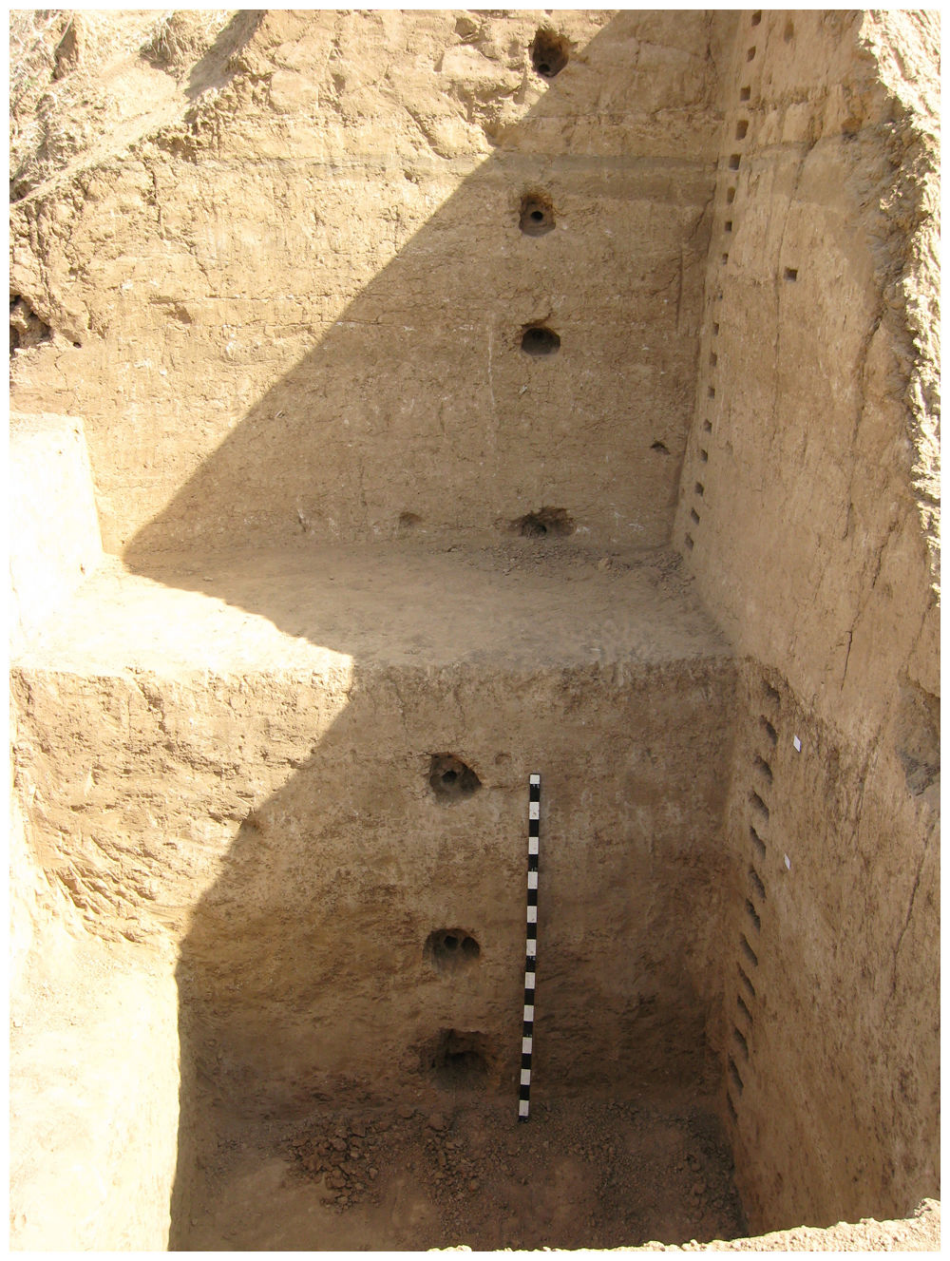
Mehtakheri excavation 2009 showing further excavation exposing Unit 3. Position of dated samples MHK 10-14: were sampled can be seen. Boundary between Unit 2 and 3 marked by change in colour due to reduced calcrete in unit 3.

The site of Mehtakheri (22° 13' 44″ N, 76° 01’37” E; Figure 3), is on the right bank of the Narmada river 2.5 km downstream of the Morttaka bridge, and about 300 m to the west of Mehtakheri village. Bedrock is exposed along a cart track between the villages of Mehtakheri and Katgarha about 15 m above the Narmada river level. This level is reached by the largest floods. Cultivated fields are 15-20 m above the flood level and 30 m above the river level. These 30 m of sediments are being actively eroded along the Narmada channel during the largest floods. Artefacts, originally within the sediments, get exposed by the erosion of the sediments. The Mehtakheri locality dated here was discovered in explorations during the 1980’s and first excavated in 1990 and 1991. In 2007 and 2009 limited excavation to obtain samples for optical dating were undertaken. In 2007 a section was scraped close to the 1990 excavation (section 2 Figure 4) and at the boundary of the preserved alluvium and gullied zone (section 3 Figure 5), the while in 2009 a fresh excavation was undertaken (Section 1 Figure 4). Samples for optical dating were collected during the 2007 and 2009 seasons. All of the sections yielded microblade assemblages. The sequence exposed and studied in sections 1-3 all rest on bedrock 15 m above the present Narmada River channel. To the east of Mehtakheri village the bedrock level goes down to the river level and 15 m of older sediments are exposed below Late Pleistocene sediments similar to those to the west of Mehtakheri village. The older sediments consist of pink calcareous silt with calcrete along the bedding planes which rests on bedrock exposed at the river level, overlain by cobbly boulder gravel in which the basalt cobbles and boulders have been strongly weathered. A single artefact, a Levallois core was recovered from the silt horizon while the surface of weathered cobbly gravel had un-abraded Middle Palaeolithic artefacts and the gravel itself yielded an assemblage of abraded large (>10 cm) quartzite flakes [49].

**Figure 6:**
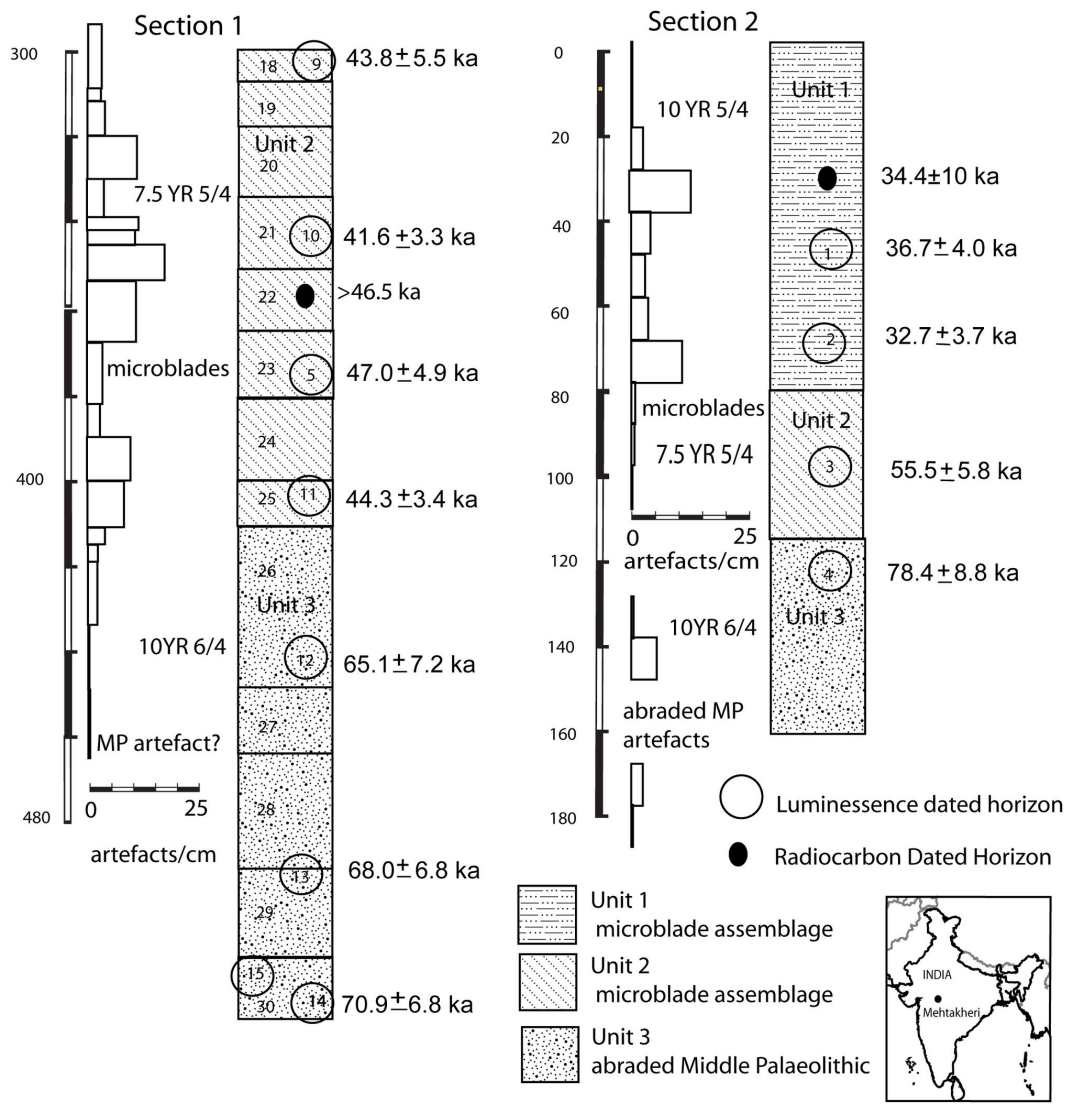
Stratigraphy and position of dated samples and artefacts in Sections 1 and 2. Artefact frequency is shown to the left of the sections.

**Figure 7:**
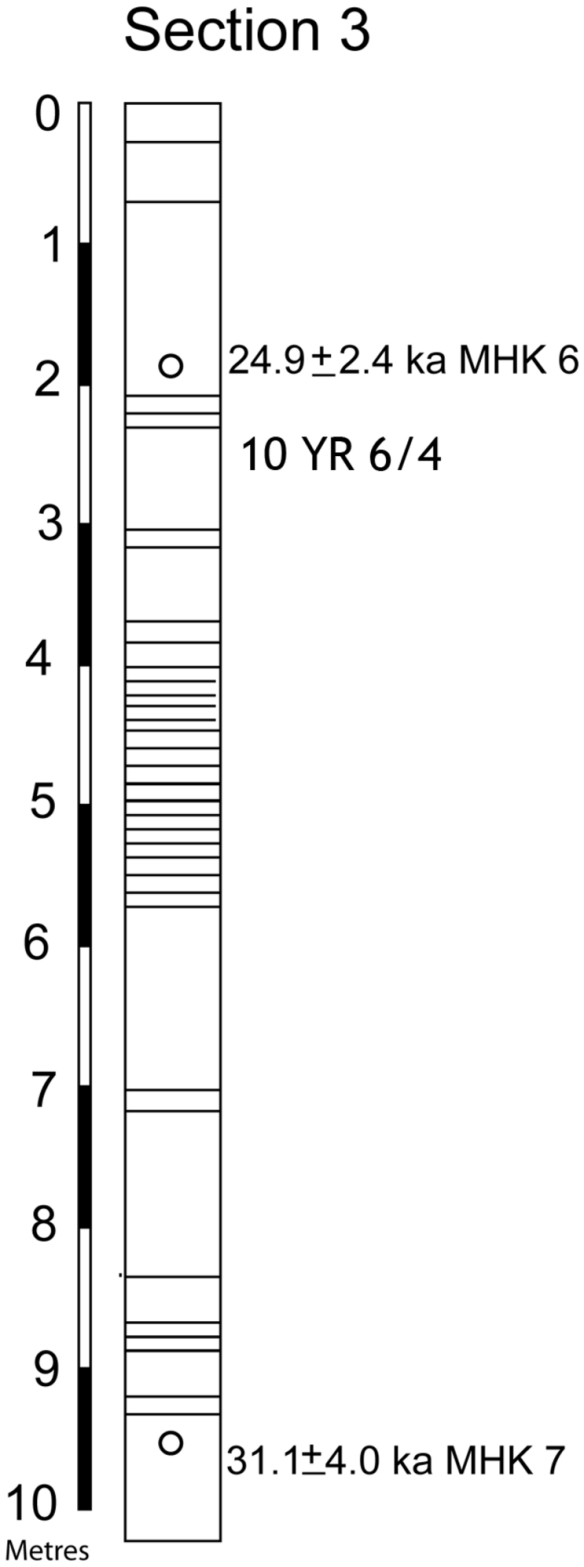
Section 3 showing the position of the dated samples. Boundaries between individual beds are shown.

**Figure 8:**
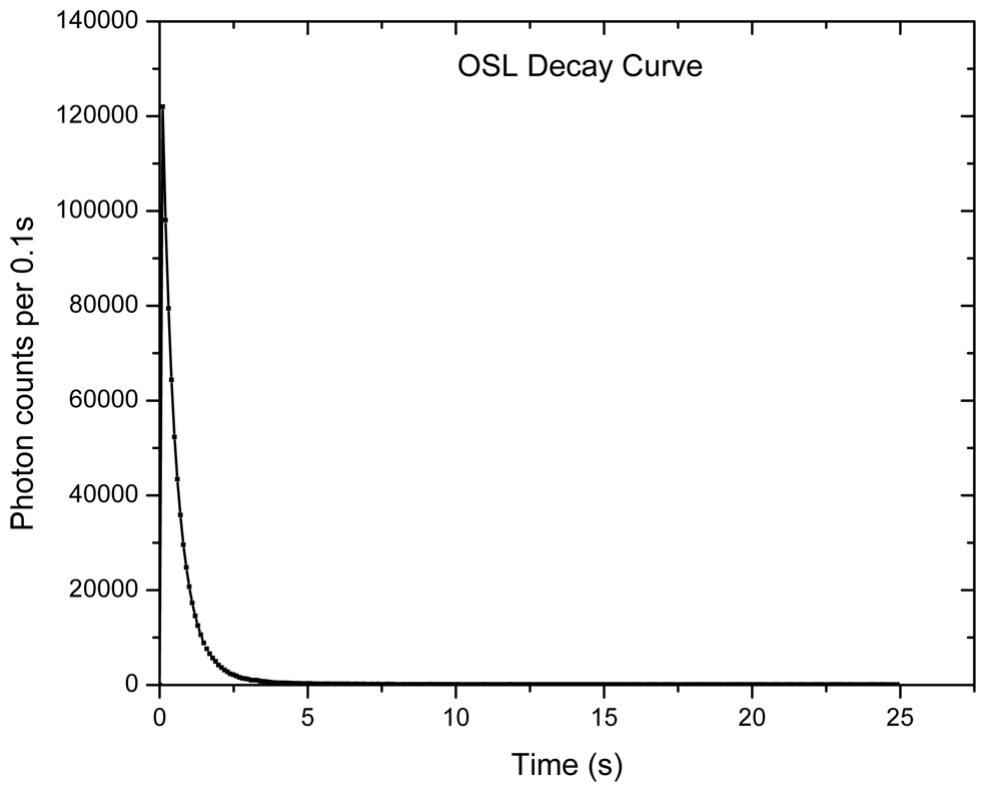
Decay curve for one of the sample.

**Figure 9:**
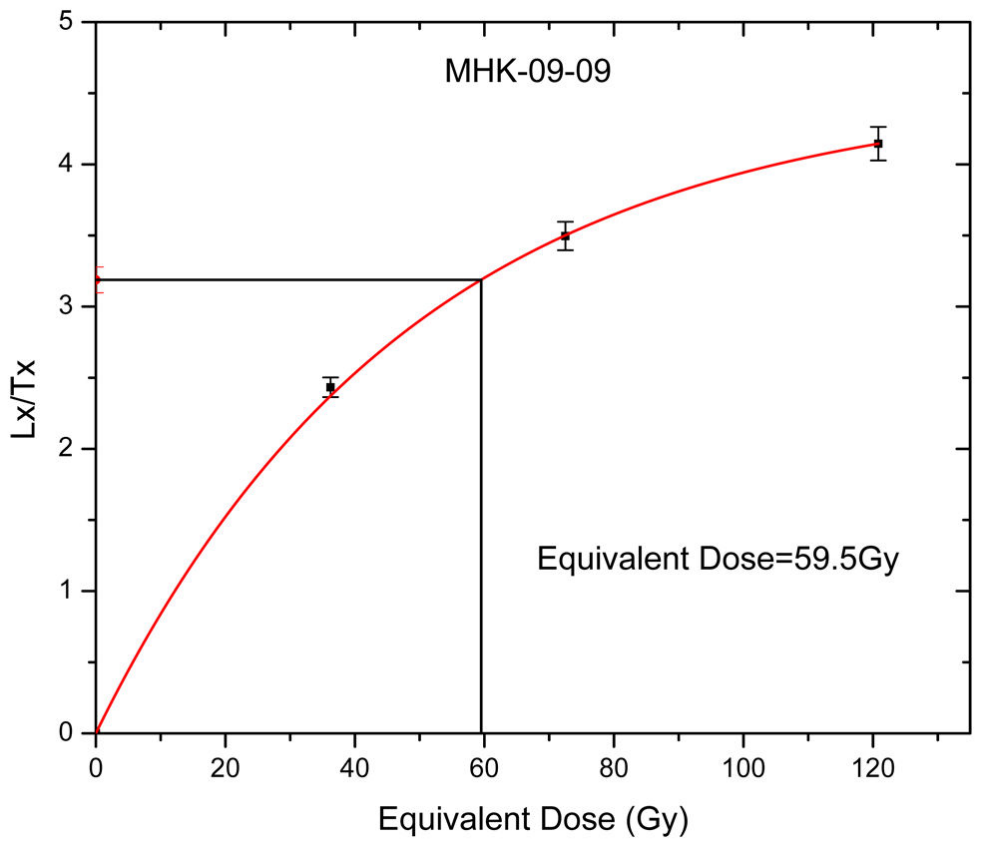
Growth curve for MHK-09-09 sample.

12 m of sediments are exposed and recorded in the three sections (Figures 6 and 7). From top to bottom, the sediments can be divided into Units 1, 2 and 3. Section 3 exposes the upper 10 m of Unit 1 with dates from its top and bottom. In Section 2 the lower part of Unit 1, containing a microblade assemblage, and the lower part of Unit 2 are exposed with an erosional contact between them. The lower part of section 2 exposes part of Unit 3. The age of the top of unit 3 in section 2 is older than that in section 1, implying an erosional contact between units 2 and 3. The erosional contact between Units 1 and 2 is clearly seen in section 1 where it is marked by a fluvial sand which cuts into unit 2 (Figure 8). The contact between unit 2 and 3 is marked by greater pedogenesis in unit 2 (Figure 9). The fluvial processes that deposited the sediments are inferred to be similar in all the three units. The sediments are sandy silts and silty sand with the sand percentage varying from 10 to 50 percent with most samples having sand in the range between 15–20 percent (Table 2). Unit 2 is similar to unit 1 and 3 in depositional processes but differs from both in having a greater degree of post depositional weathering and pedogenesis. Microblade assemblages are present in Unit 1of section 2 and Unit 2 of section 1. A few artifacts in upper part of the underlying unit in both sections are considered to be part of the overlying archaeological assemblages.

**Table 2:** Percentage of sand in samples from Section 1 Mehtakheri.

Depth in Cm	Layer no	%Sand	Depth in Cm	Layer no	%Sand
10	1	15.5	300	17	24.5
20	2	20.5	310	18	53.5
30	2	22.0	320	19	30.0
40	2	20.5	330	21	9.5
50	3	20.5	340	21	20.5
60	3	12.0	350	22	24.5
70	3	11.5	360	22	10.0
80	3	31.5	370	23	15.5
90	4	18.5	380	24	15.0
100	4	23.5	390	24	15.0
110	5	9.0	400	25	18.5
120	5	14.0	410	26	24.5
130	6	10.5	420	26	19.5
140	6	10.5	430	26	19.0
150	7	16.5	440	26	
160	7		450	26	26.5
170	7	17.0	460	27	
180	7	15.5	470	28	26.
190	7		480	28	26.5
200	8	10.0	490	29	13.5
210	8	15.0	500	29	
220	9	9.0	510	29	12.5
230	9	13.0	520	29	17.0
240	9	6.5	530	30	17.0
250	9	9.5	540	30	6.5
260	9	18.0	550	30	8.5
270	13	14.0	560	30	25.0
280	14	3.5			
290	15	1.5			

### Optical Dating of the Mehtakheri site

Figures 8 and 9 show the sampling of Section 2. The section was draped in thick black cloth and the samples were collected in custom designed aluminum pipes [50] and processed under dark room conditions of subdued red light. The outer 3 cm thick layer was removed and used for dose rate determination. The interior of the sample was treated with 1N HCl and followed by H_2_O_2_ to remove the carbonate and the organic matter respectively. All the samples had high carbonate content. The samples were washed, dried and sieved to get 90-150 µm grain fractions. The grain fractions were then treated with excess 40% HF for 80 minutes to remove the outer 20 µm alpha skin and to dissolve feldspars. The quartz was separated using a Frantz magnetic separator at a magnetic field of 10K gauss. The purity of quartz grains was analyzed using their infrared stimulated luminescence signal (IRSL), and any sample depicting finite IRSL signal was re etched and retested.

**Figure 10:**
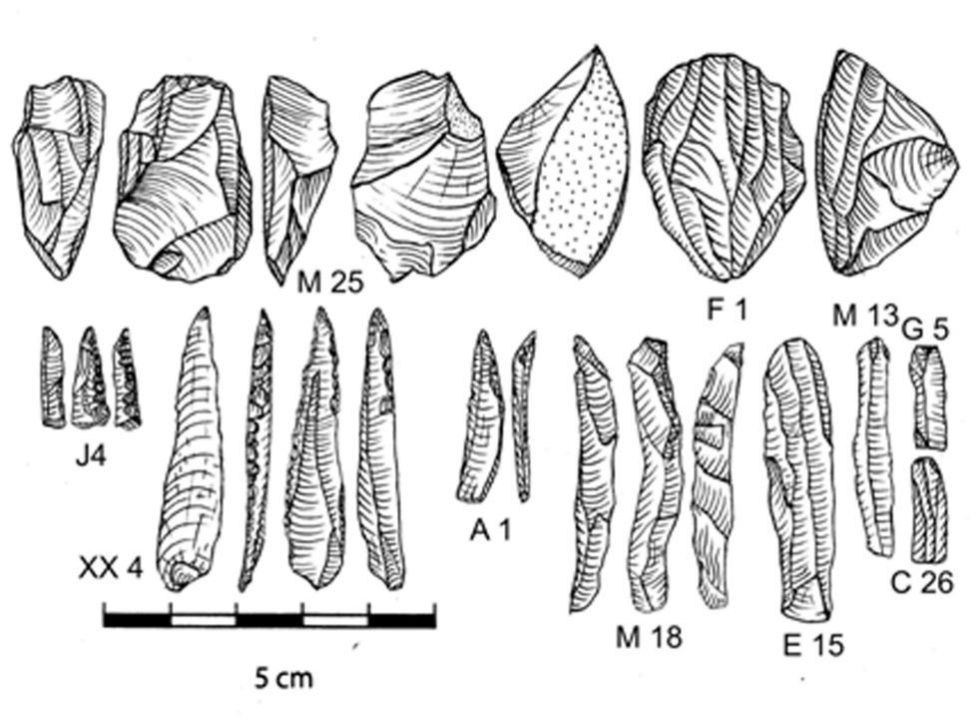
Microblade industry from Mehtakheri: M 25and F 1: Microblade cores; J4, XX4, A 1: backed blades; M 18: crested guiding ridge flake, E 15 and M 13 blades; G 5 & C 26 broken blades. XX4 is a complete example of J4. J4 is from the excavation while XX4 is from surface collection close to the excavated section.

**Figure 11:**
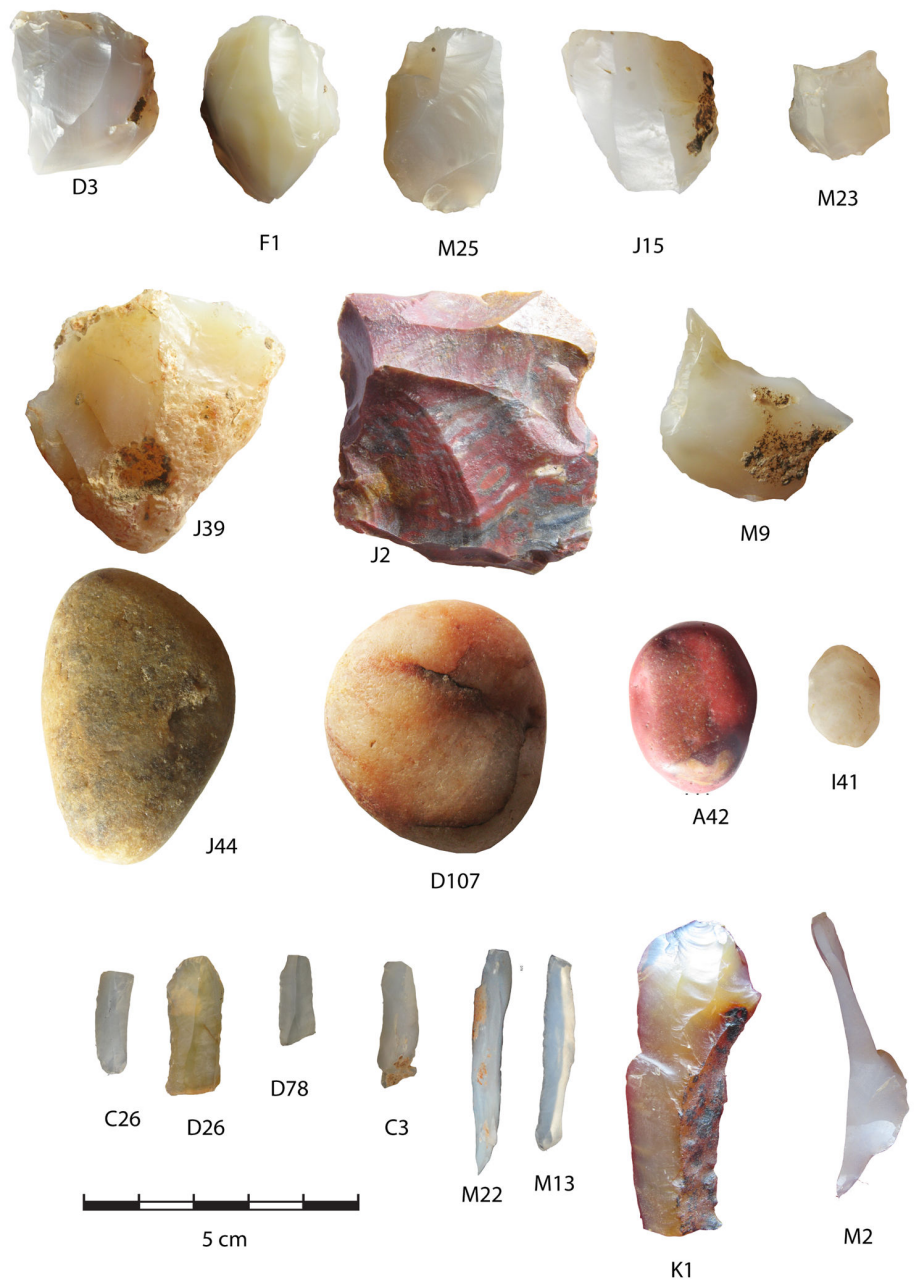
D3, F1, M25, J15 and M23 are microblade cores. J39 is a trimmed nodule. J2 is is a retouched flake on multicoloured chert and M9 is a perforator made on a platform rejuvenation flake. J44, D107, A42 and I41 are hammerstones of various sizes. C26, D26, D78, C3 are broken flakes and M22 and M13 complete flakes. K1 and M2 are from the initial stages of core reduction showing the much larger initial size of the cores. M 2 also retains a part of the crested guiding ridge.

OSL measurements were carried out on a Risoe TL–OSL reader with detection in a UV window defined by a filter combination of Hoya-U340 and Schott BG-39. The paleodose was estimated using Single Aliquot Regeneration procedure [51]. The analysis conditions were, 1) preheat of 220^°^C for 10s, 2) cut heat of 200°C 3) recycling ratios 1±0.1, 4) early light subtraction and 5) dose recovery of ±10%. The De showed a preheat plateau in the range 200-250^°^C. Typical shine down curve (Figure 10), SAR growth curve (Figure 11) are shown. A mean of De’s from individual aliquots was used to estimate the age.

The concentration of radio-elements U and Th was measured using ZnS (Ag) thick source alpha counting and NaI (Tl) gamma ray spectrometry was used to estimate the K. These were used to compute the annual dose rate. The water content was measured on the samples as received. The dose rate computations assumed a radioactive equilibrium.

Unit 1 was dated in sections 2 and 3 by sample numbers MHK 1, 2, 6, 7 (Table 3 Figures 6 and 7) and a radiocarbon date. Unit 2 was dated in sections 1 and 2 by samples 3, 5, 9, 10, and 11 and a radiocarbon date (Table 3 Figure 6). Unit 3 was dated by sample nos. 4, 13 and 14. The upper part of Unit 1, exposed in section 3 has an optical age of 24.9±2.4 ka (MHK 6) at the top and 31.1±4.0 (MHK 7) at the base. Artifact bearing horizons at the base of unit 1 in section 2 gave optical ages of 36.7±4.0 ka (MHK 1), 32.7±3.7 ka (MHK 2) and a radiocarbon date on mollusk shell of 34,380 ± 991 cal a BP, (30,106+1040-920, A 6518) was also available. Unit 3 has optical ages of 65.1 ± 7.2 ka (MHK 12), 68.0± 6.8 ka (MHK 13), 70.9 ± 6.8 ka (MHK 14) and 78.4 ± 8.8 ka (MHK 4) (Figure 3). Optical ages of artifact bearing horizon (Unit 2 in section 1), are 47.0±4.9 ka (MHK 5) 43.8±5.5 ka (MHK 9) 41.6±3.3 (MKH 10) 44.3±3.4 ka (MHK 11) and give a weighted mean of 44.2 ka at 1 σ level. A date of 55 ka for unit 2 from section 2 has not been used in these calculations as this date implies it comes from part of unit 2 which was not sampled in section 1 and no artefacts were certainly associated with it. The dates from unit 2, unlike unit 1 and 3 are considered to underestimate the true age somewhat, for the following reasons:

**Table 3:** Optical Dating of Samples from Mehtakheri.

**Sample no**	**no. of aliquots**	**Dose (Gy)**	**U (ppm)**	**Th (ppm)**	**K (%)**	**CR (µGy/a)**	**Water Content**	**DR (Gy/ka)**	**Age (ka)**
**Samples from Unit 1**									
**MHK-07-06**	40	35.74±0.07	1.98±0.37	3.82±1.26	0.83±0.08	148.5	0.15	1.43±0.14	24.9±2.4
**MHK-07-07**	39	43.05±0.10	2.05±0.70	3.90±1.28	0.87±0.07	49.2	0.15	1.39±0.18	31.1±4.0
**MHK-07-01**	37	46.00±0.20	1.44±0.38	5.27±1.31	0.74±0.08	55.3	0.15	1.25±0.14	36.7±4.0
**MHK-07-02**	31	51.43±0.09	1.92±0.56	6.79±1.92	0.91±0.06	53.7	0.15	1.57±0.18	32.7±3.7
**Samples from Unit 2**									
**MHK-09-09**	38	57.62±0.61	1.42±0.23	4.02±0.81	0.92±0.17	52.1	0.15	1.32±0.17	43.8±5.5
**MHK-09-10**	43	58.86±0.68	1.90±0.27	3.07±0.94	1.00±0.06	50.4	0.15	1.41±0.11	41.6±3.3
**MHK-07-05**	36	80.34±0.17	1.51±0.50	7.68±1.73	1.12±0.09	43.5	0.15	1.71±0.18	47.0±4.9
**MHK-09-10**	46	67.92±0.80	1.82±0.27	3.84±0.94	1.11±0.07	46.5	0.15	1.53±0.12	44.3±3.4
**MHK-07-03**	39	72.47±0.20	1.47±0.40	4.63±1.37	0.85±0.06	52.4	0.15	1.31±0.14	55.5±5.8
**Samples from Unit 3**									
**MHK-09-12**	51	69.36±0.77	1.36±0.27	3.62±0.95	0.67±0.09	41.9	0.15	1.07±0.12	65.1±7.2
**MHK-09-13**	32	82.19±1.36	1.38±0.33	4.44±1.16	0.78±0.06	41.9	0.15	1.21±0.12	68.0±6.8
**MHK-09-15**	30	85.65±1.23	1.70±0.29	3.15±1.04	0.80±0.07	40.6	0.15	1.21±0.12	70.9±6.8
**MHK-07-04**	33	86.94±0.47	1.56±0.31	3.42±1.08	0.68±0.09	50.6	0.15	1.11±0.13	78.4±8.8

Samples have been arranged in stratigraphic order from uppermost to lowermost. The measurements were made on quartz extracts using the standard single aliquot regeneration dating protocols comprising a five point regeneration scheme using the UV emission of quartz. Aliquots with recycling ratio of 1 0.1 were used. The radioactivity was measured using gamma ray spectrometry and alpha counting. Samples are arranged in their stratigraphic order.

1. In Unit 1 there is good agreement between the optical and radiocarbon dates whereas the radiocarbon date from unit 2 implies that the optical date has underestimated the true age of the unit. Unit 3 is beyond the limit of radiocarbon dating.
2. Optical dates imply a sedimentation rate for both unit 1 and 3 of ~1m/ka while it for unit 2, it is 0.12/ka, an almost a tenfold decrease. The depositional processes in all the units are similar, so the sedimentation rates should also be similar. The apparent decrease in the sedimentation rate therefore reflects the greater dispersion of the optical ages in Unit 2 compared to Units 1 and 3 due to effects of pedogeneis on the estimates of dose rate, rather than a longer duration for the deposition. If the sedimentation rate for unit 2 was similar to that of units 1 and 3 then, 1.2 m of Unit 2 represents a time interval of 1-2 ka rather than almost 10 ka implied by the dispersion of the dates.
3. Pedogenesis in Unit 2 has led to local scale micro-dosimetric variations that in turn resulted in an apparent increase in the dose rate of unit 2 compared to unit 1 and 3 by about 20% (Table 3). This implies that the ages in unit 2, derived using present day radioactivity could be underestimated and assuming a linear increase of dose rate with time, this underestimation in age could be ~10%. In this case the average age of the microblade technology would be ~ 48 ka ± 2 ka.
4. This older age estimation for unit 2 reduces the duration of the gap in sedimentation between units 2 and 3 from >15 ka to 6-7 ka which seems reasonable.
5. A radiocarbon date of > 46,555 cal BP (>42,900, AA8463) from this unit further supports the inference that the optical ages for unit 2 are underestimated as only the oldest of the four optical ages is older than the infinite radiocarbon date.
6. The oldest date in unit 2, MHK 5, comes from a horizon with a low artefact frequency and the youngest date MHK 10 comes from a horizon with high artefact frequency. It is likely that the artefact horizons coincide with breaks in sedimentation when soils would have developed and so pedogenesis would be higher in the intervals with high human activity and lower in the units with low human activity. Thus the sample with low pedogenesis gave the oldest date and the sample with high pedogenesis, gave the youngest date which further supports our inference that pedogenesis is leading to underestimation of the age.
7. The oldest date, the date calculated taking into consideration the effect of pedogensis on dose rate estimation, and the radiocarbon date are all in agreement with an age estimate of ~48 ka.

Thus we consider that oldest of the optical dates from unit 2 should be the closest to the true age.

The oldest date (MHK 07-4) from unit 3 in section 2 of 78.4 ± 8.8 ka is younger than the Middle Palaeolithic on the surface of the gravel to the east of Mehtakheri and also the Levalloisian core from the underlying silt. Severely abraded Middle Palaeolithic artefacts occurred in lag surfaces in the lower part of Unit 3.

### Microblade assemblage from 2009 excavation

The microblade assemblage of Unit 2, excavated in 2009, comprises microblades, microblade cores and backed blades (Figures 10 and 11). The excavated assemblage includes two backed blades, 50 complete blades, 100 broken blades, 28 chunks, 10 microblade cores, 634 flakes, 5 hammerstones, 5 nodules, 11 pebbles, two retouched flakes (Table 4). Rubble (52 pieces), and shells occur along with the artifacts. Most of the artifacts belong to the microblade *chaine operatoire*. A second *chaine operatoire* related to flake production is represented by retouched flakes which were introduced as finished tools into the assemblage

**Table 4:** Artifact types from Mehtakheri 2009.

**Type**	**number**	**%**	**Average** **cm**	**Max size cm**	**Minimum size cm**	**%chalcedony**
**Nodule/trimmed nodule**	5	0.52	4.27	5.44	3.06	80
**Hammerstone**	5	0.52	4.9	7.12	2.71	20
**Pebble**	11	1.26	3.95	10.82	0.8	18
**Rubble**	52	5.79	2.24	7.21	.63	20
**Retouched Flakes**	2	0.22				50
**Backed blade**	2	0.22	2.6			100
**Microblade**	50	5.57	2.8	4.72	1.1	84
**Broken Microblade**	100	11.15	1.5	3.7	0.1	97
**Microblade Cores**	12	1.34	2.8	3.43	1.84	84
**Chunk**	28	3,12	2.2	4.16	0.76	90
**Flake**	634	70.48	1.76	6.93	0.42	91
**Total**	901	99.92				

The assemblage consists of three components, one related to microblade production, the second related to flake production and the third to stone used for purposes other than stone tool making. The microblade component of the assemblage is made exclusively on chalcedony and chert with chert being a minor component. Chalcedony is mostly in the form of nodules from basaltic bedrock outcrops close to the site. Most of the chalcedony artifacts belong to the microblade *chaine operatoire*. The flake production was on quartzite. Quartzite was obtained from river gravels adjacent to the site. In the 2009 excavation no quartzite flake cores were found. The surface collection and earlier excavations however have yielded some quartzite cores [52]. Quartzite cores are single direction cores on flat pebbles. Abraded large flakes from pre-microblade contexts were also selected as cores. A few radial cores were also found. The flakes from the quartzite *chaine operatoire* occasionally had evidence of secondary modification. In the 2009 collection there are some quartzite flakes which might have been used as tools, but they did not show any secondary modification.

The two retouched flake tools are not made on quartzite. The first is a scraper on chert (Figure 10: J2 and the second a perforator made on a chalcedony platform rejuvenation flake (Figure 11: M9). Most of the quartzite in the assemblage is "rubble". Rubble is produced by human activity as it is found exclusively in the horizons with artifacts but the pieces are chunky, and although they have breakage surfaces they lack clear striking platforms and bulbar scars. Further study is needed to clearly distinguish between the use of stone for tool production and other activities. Pounding, grinding and heating of rocks are possible alternate use of stone in the assemblage. Some basalt fragments were also found in the excavation and appear to be part of the non stone tool component of the stone assemblage. As the natural sediments at the site are all sand or smaller in size, all the stone material is introduced by human activity, allowing us observe the introduction of stone for purposes others than tool production.

**Figure 12:**
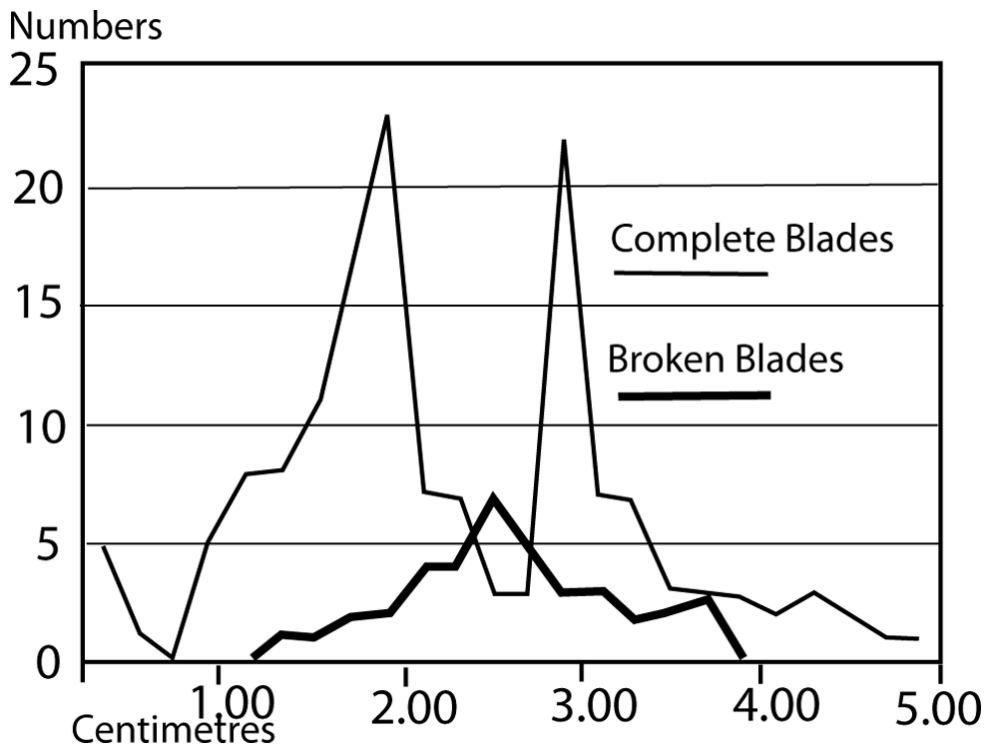
Size distribution of broken and complete blades from Mehtakheri 2009.

**Figure 13:**
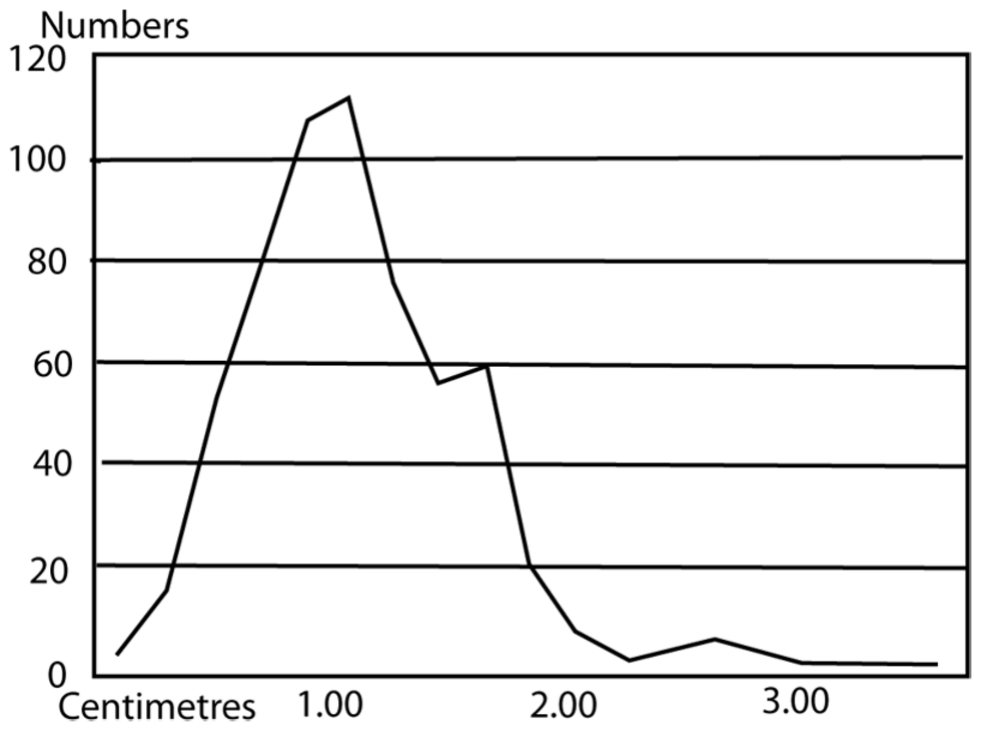
Size distribution for flakes from Mehtakheri 2009.

Two backed blades are the only "shaped" products of the microblade *chaine operatoire*. Microblades are considerably more common with twice as many broken microblades (100) as complete ones (52). Although the high level of microblade breakage could be due to their fragility, it is possible that they were deliberately broken to fit into the hafts. The equally fragile flakes are rarely broken. As can be seen in Figure 12 the broken blades fall into two modes on either side of the complete blade mode. The unbroken blades therefore were not broken as they conformed to the target size, while blades longer than this size were broken to conform to it. The average size of the cores is the same as the average size of the blades (Table 4), indicating that many blades are longer than any of the microblade cores. It also implies that the "target size" of the blades was the size of the smallest blades removed from the cores. Most of the microblade cores are 2-3 cm in maximum dimension. The ratio of 10 cores to 150 complete/broken microblades, and over 600 flakes also indicates extensive reduction of the cores and/or export of cores from the site. Inspite of the large number of flakes, only two flakes have completely cortical dorsal surfaces and 70% of the flakes have no trace of cortex at all. The remaining 30%, with some cortex, usually have very small traces of cortex. The size of the flakes is also quite small with the average size being 1.7 cm and the modal size 1.0-1.2 cm (Table 4 Figure 13). The assemblage therefore lacks the initial stages of core preparation and is mainly related to microblade production and reshaping of cores to continue microblade production. Cores appear to have been curated, and the discarded material is the debitage from core maintenance or core preparation. If blades were deliberately broken then the broken blades might be related to replacement of the cutting edges in hafted tools. The cores were prepared by removal of the cortical portion of the original nodule from the striking platform and the surface from which the microblades were to be detached. The blades were probably detached by hard hammer technique and quartzite hammer stones are present in the assemblage. A few crested guiding ridge flakes occur in the assemblage, as do two platform rejuvenation flakes. One surprising finding was clear battering marks on very small pebbles (1-2 cm maximum dimension) showing that hammer stones in a range of sizes were used (Figure 11: I41). Although river gravel is available close to the site, most of the raw material is obtained as nodules of secondary minerals derived from basalt outcrops. The angle between the striking platform surface and the blade detachment surface is around 70 ^°^. In spite of exhaustive exploitation of the cores, in most cases some cortex remains. This cortex is usually on the right side of the core if the striking platform surface is held proximally and the exploited surface uppermost. The cores are roughly pyramidal in shape, although extensive exploitation or initial shape of the nodule could result in flat cores.

## Discussion and Conclusions

Microblade technology appears for the first time in South and East Africa around 60-65 ka in the Howieson’s Poort assemblages from South Africa [21,22,23], 64-57 ka at the Mumba rock shelter [31] and 62 ± 5 ka from the Naisiusiu beds in East Africa [30]. Although an even earlier date of 71 ka for microblades has recently been obtained from Pinnacle Point in South Africa [53] extending the origin of the technology, this microblade technology does not continue after 57-60 ka in either South or East Africa. The precision and the antiquity of the dates published for Howieson’s Poort has also recently been questioned [22,24]. An origin for the Indian microblades from the Howieson’s Poort has been suggested by Mellars [35] which would require it to disperse to Indian before or around 60 ka, before it disappeared from Africa. It is interesting that the site of Tarasma in the Nile valley also has evidence for the brief presence of blade technology around 60 ka [32]. It is also noteworthy that while the Indian microblade assemblages are not closely related to any entity outside of India, its closest match is with sites dating to around 60 ka in Africa. The dates for these early industries in Africa are themselves not precisely constrained due to the limitations of the dating technology.

Evidence for modern humans during MIS 5 occurs in the Levant [54], the Arabian Peninsula [26,27,55,56], China [56] and Southeast Asia [57]. The stone tool technology associated or attributed to modern humans during MIS 5 is not easily distinguished from that used by archaic humans [17,58],. In India, MIS 5 contexts are associated with the Middle Paleolithic [59,60] having roots in the Acheulian. Projectile [61] and blade technology, which have early but discontinuous appearances in Africa [9,11] and Western Eurasia [10,12] are not a part of the Acheulian or Middle Paleolithic in India. The Indian MIS 5 assemblages therefore are most probably associated with Indian archaic humans and the Middle Paleolithic in India has little or no similarity to the Middle Paleolithic reported from MIS 5 contexts in Arabia. The Arabian tools have resemblances to Africa [26,45] and the Levant [46].

The Southeast Asian record shows a marked cultural boundary with the Indian Subcontinent throughout the Late Pleistocene, with similarities to Southern China rather than India. Recognition of this difference has been hampered by the mistaken identification of Late Pleistocene flake and core industries in Southeast Asia as Lower Palaeolithic Chopper Chopping tools industries [62]. It has been shown that the Indian representative of the “chopper chopping” tool group, the Soanian [63], is derived from Late Pleistocene sediments, while Acheulian artefacts are found where Lower and Middle Pleistocene sediments are exposed [8,63,64]. A similar situation exists in Southeast Asia where the site of Kota Tampan, the type site for the Malaysian chopper chopping tool industry [62] has been associated with the Youngest Toba Tephra (YTT), dating to 73.88 ± 0.32 ka [65]. These Late Pleistocene chopper chopping tool assemblages, now usually called core and flake assemblages, are now considered to be associated with modern humans [66]. Modern human fossils are dated to 34-41 ka at Niah cave [67] and 51-46 ka at Tam Pa Ling, Laos [68] which are some of the oldest modern human skeletons outside Africa. While these dates are close to the second phase of modern human dispersals, considerable evidence exists to indicate that modern humans with core and flake technology occupied Southeast Asia and parts of Eastern Asia much earlier. As already mentioned the Kota Tampan artefacts are below the YTT and are now considered to be the work of modern humans [66]. In Java, the Punung fauna has been dated to the MIS 5 [57]. This fauna represents a significant ecological shift from a more grassland adapted fauna associated with *Homo* *erectus* to a fauna including species typical of a rainforest biome. Modern humans may be part of this fauna [69]. Zhirendong [56], in Southern China has yielded remains of a possible modern human below stalagmite layers dated to ~ 100 ka, and is also associated with rainforest adapted species such as the orang-utan. Thus while more data is needed, it seems possible that modern humans entered Southeast Asia during a period of ecological change. It has also been suggested that the rainforest ecozone was not occupied by *Homo* *erectus* [70,71] so that some of these areas might have been colonized by humans for the first time.

The contrast between the technology associated with modern humans in the Indian Subcontinent and Southeast Asia is present right from the earliest presence of modern humans in the two regions thus making it more likely that modern humans reached Southeast Asia from Southern China rather than that the differences emerged after modern humans reached Southeast Asia from India. The variation in the degree of Neanderthal and Denisovan ancestry in present day populations can also be explained by an earlier dispersal of modern humans to SE Asia via China rather than the Indian subcontinent. Denisovan ancestry is significant in Island but not mainland SE Asia [72]. This is explained if populations in Island SE Asia are descended from populations which spread through China when Denisovan populations were still present. Present day Chinese have different and greater amounts of Neanderthal ancestry than populations elsewhere [73]. but lack significant Denisovan ancestry [72]. This could be due to admixture with Neanderthals in Central Asia when modern humans expanded from there into China during MIS 3 times after Denisovians had become extinct.

After the dispersal of *Homo* *erectus* , genetic, cultural and biological differentiation of human populations occurred over the large hominin range. In spite of this differentiation, evidence of admixture of genes from at least two archaic human populations into present day modern humans has been identified [72,73,74,75,76],. 

Based on our older ages for microblade technology in the Indian Subcontinent, and the arguments presented above, we present the following model for modern human dispersal (as illustrated in Figure 2):

1. During MIS 6 widely dispersed hominin populations with a common ancestor in *Homo* *erectus* had differentiated into distinct populations with modern humans and possibly other archaics in Africa, Neanderthals in Europe, Denisovans in temperate Eastern Eurasia and archaic Indians in the Indian Subcontinent and Sundaland (Figure 2.1).
2. During the Interglacial climate of MIS 5, modern humans expanded into Eurasia at the expense of both Neanderthals and Denisovans and reached SE Asia. Due to competition with Indian archaics, modern humans were unable to disperse into the Indian Subcontinent and modern humans reached SE Asia at this time via a northerly route through the Middle East, Central and Eastern Eurasia and Southern China. During this time admixture with both Neanderthals and Denisovans is likely to have occurred in the modern humans reaching SE Asia (Figure 2.2)
3. During the glacial climate of MIS 4 temperate adapted populations such as Neanderthals expanded into the Middle East along the Mediterranean coast and into Iran and Central Asia. The desert belt of Northern Africa and Arabia was abandoned. Competition between modern humans and Archaic Indians was intense and modern humans were able to disperse into the Indian Subcontinent. It is possible that Neanderthals expanded at the expense of Denisovans during this time (Figure 2.3).
4. During the interglacial climate of MIS 3 modern humans dispersed from India into adjoining regions. Modern human populations in SE Asia and parts of Africa might also have expanded at this time. The last archaics disappear (Figure 2.4).

Sharp cultural boundaries between the Indian Subcontinent and adjacent areas are not in conformity with a rapid dispersal through India to SE Asia from Africa. Archaeological sites in Arabia and Africa dating to MIS 5 show some distinctive technological features, such as Nubian cores and bifacial projectiles which are absent from any assemblage in the Indian Subcontinent, making it unlikely that modern humans dispersed into the Indian Subcontinent at this time. On the other hand microblade technology or blade technology is attested to at archaeological sites dating to around 60 ka in Africa which have a closer resemblance to the Indian microblade technology. Core and flake assemblages are associated with modern humans in SE Asia and may date back to MIS 5 times. We suggest that the later entry of modern humans into the Indian Subcontinent compared to adjacent regions is because Indian Archaics could easily compete with modern humans during climate conditions favorable to both. Although the impact of climate and competition on modern human dispersals has been considered extensively, we believe that the differing nature of competitive interactions between modern humans and archaic populations adapted to different environmental conditions is a crucial factor, especially in relation to the Indian Subcontinent the major region outside Africa with tropically adapted populations. This factor is considered for the first time here.
